# Allogeneic hematopoietic cell transplantation in patients ⩾70 years: which patients may benefit?

**DOI:** 10.1038/bcj.2016.54

**Published:** 2016-07-08

**Authors:** S P Haen, M Pham, C Faul, D Dörfel, W Vogel, L Kanz, W A Bethge

**Affiliations:** 1 Department for Oncology, Hematology, Immunology, Rheumatology and Pulmonology, University Hospital Tuebingen, Tuebingen, Germany; 2 Department for Immunology, Interfacultary Center for Cell Biology, Tuebingen, Germany

Allogeneic hematopoietic cell transplantation (HCT) can be the only curative treatment option for patients with hematologic malignancies. Although in younger patients (that is, patients aged ⩽60 years) allogeneic HCT has been performed for decades. The development of reduced intensity conditioning (RIC) has enabled HCT in elderly patients establishing that age is no independent risk factor for HCT outcome.^1, 2^ This may be of major therapeutic impact, as most hematologic malignancies peak in incidence above 60 years of age^3^ and prognosis with conventional treatment is dismal in this age group.^4^ Moreover, HCT in elderly patients represents a challenge due to age-related problems such as comorbidities, pre-treatment and increased incidence and lower tolerability of acute graft versus host disease (aGvHD).^5^ RIC produces less pronounced antiproliferative effects with a higher risk of relapse but preserves immunological antileukemic activity.^6^ RIC-based HCT of patients ⩾60 years results in limited toxicity, favorable engraftment and survival.^2^ However, very limited evidence is available for HCT in patients with even more advanced age, that is, patients ⩾70 years. Here we report our experience in 56 consecutive patients aged ⩾70 years (Table 1) undergoing allogeneic HCT between 2005 and 2015. No patients >70 years had been transplanted before 2005 at our center. The median comorbidity index (HCT-CI)^7^ was 1 point (0–10 points) with a median integrated non-relapse mortality (NRM) score^8^ of 5 points (2–12 points). Disease risk stratification^9^ at transplantation was low risk in 19, intermediate risk in 10, high risk in 13 and very high risk in 14 patients.

Median time between diagnosis and HCT was 5 months (1–190 months) with the longest in one patient each with chronic lymphoid leukemia, non-Hodgkin lymphoma and primary myelofibrosis (PMF) (63, 42 and 190 months, respectively). All patients received RIC (Supplementary Table 1) followed by transplantation of a median of 6.54 × 10^6^ CD34^+^ cells per kg body weight (1.95–18.04 × 10^6^) of granulocyte colony-stimulating factor (Lenograstim, Chugai Pharma, Frankfurt, Germany)-mobilized peripheral blood stem cells (*n*=55) or unmanipulated bone marrow (*n*=1) from high-resolution human leukocyte antigen (HLA)-typed (HLA-A, -B, -C, -DRB1 and -DQB1) donors (matched related (*n*=7; 13%), matched unrelated (*n*=37; 66%) or mismatched unrelated donors (*n*=12; 21%). At HCT, disease status was complete remission (CR), partial remission (PR) or active disease (AD) in 23, 15 and 18 patients, respectively. GvHD prophylaxis was performed with standard protocols (Supplementary Table 1).

Median neutrophil (>500/μl) and platelet engraftment (>20 000/μl) was on days 19 (days 9–43) and 15 (days 10–-398), respectively. Four patients died prior hematopoietic regeneration between days 11 and 22 due to complications (infection *n*=3 and hemorrhage *n*=1).

Kaplan–Meier (SPSS V.22, 2013, IBM, Armonk, NY, USA) estimate (Table 1) for median overall survival (OS) was 18.0 months (0.4–123.9 months). One-, 2- and 3- year OS was 54.7%, 46.1% and 42.8%, respectively. At the end of follow-up, 29 patients had died (52%). Causes of death were relapse (*n*=20), infection (*n*=3), GvHD (*n*=2), hemorrhage (*n*=2), embolism (*n*=1) and graft failure (*n*=1). Cumulative incidence of NRM was 9.3%, 18.1% and 18.1% at day 100, 12 and 24 months, respectively (Supplementary Figure 1).

Median disease-free survival (DFS; Supplementary Figure 1) was 8.4 months (0.4–123.9 months). One-, 2- and 3-year DFS was 46.0%, 43.1% and 39.5%, respectively. Tenty-two patients (39%) experienced relapse at a median of 3.9 months after HCT (0.8–44.6 months). Cumulative incidence of relapse was 8.7%, 34.7% and 45.6% at day 100, 12 and 24 months, respectively (Supplementary Figure 1).

Sixteen patients (29%) developed aGvHD with a median grade 1 (grades 1–4).^10^ Grade 2, 3 and 4 aGvHD were observed in 5, 3 and 1 patients, respectively. Chronic GvHD (cGvHD) was observed in 18 patients (32%) with limited and extensive disease in 13 (23%) and 5 (9%) cases, respectively.

Patients were stratified for HCT-CI, sex, disease risk, blood group and cytomegalovirus (CMV) mismatch, duration between diagnosis and HCT, donor–recipient pairs, disease status at HCT and immunosuppression (Figure 1, OS Supplementary Table 2 and DFS Supplementary Table 3). Risk factors for survival were evaluated in univariate comparisons (Cox regression). In line with previous findings^8^ comorbidities did not influence OS (12.3 vs 43.9 months, *P*=0.83) with a tendency to favorable OS for male vs female patients (10.5 vs 50.9 months, *P*=0.06), patients with <6 months between diagnosis and HCT (26.4 vs 6.9 months, *P*=0.06) and patients with unrelated donor (UD; 19.4 vs 5.8 months, *P*=0.07). A significantly better OS was observed for patients with high-risk disease status as compared with intermediate or very high risk (70.4 vs 7.0 months, *P*=0.003). Interestingly, patients with CR at HCT did not have a better OS as compared with patients with PR or AD (41.2 vs 12.3 months, *P*=0.30). All other factors did not influence OS. Of note, patients not receiving Alemtuzumab exhibited a significantly better survival (19.4 vs 0.4 months, *P*=0.003), but only two patients received Alemtuzumab.

A better DFS was observed for patients with CR as compared with PR/AD (38.2 vs 7.5 months, *P*=0.05), as well as patients with high-risk as compared with intermediate- or very-high-risk disease (70.4 vs 4.4 months, *P*=0.002). Interestingly, a significantly better DFS was observed for patients receiving grafts from UD (16.1 vs 2.9 months, *P*=0.004). A positive impact on survival—albeit not statistically significant—was observed in patients receiving ciclosporin A (38.2 vs 6.9 months, *P*=0.09). As for OS, a significantly better DFS was observed for patients not receiving Alemtuzumab (9.8 vs 0.4 months, *P*<0.001). All other factors did not influence DFS.

At the end of follow-up, 27 patients (Supplementary Table 4) remained alive (5 women: 19% of the female population; 22 men: 81% of the male population). Underlying diseases were acute myeloid leukemia (AML) (*n*=22), myelodysplastic syndrome (MDS) (*n*=2) and PMF (*n*=3) representing long-term survival of 48% of AML, 40% of MDS and 100% of PMF patients. Two of the surviving AML patients (9%) experienced relapse (7.5 and 44.6 months after HCT, respectively) and were treated with cytoreductive therapy. One patient with PMF (33%) had disease relapse 2.9 months after HCT. Hence, 24 patients (43%) of the whole cohort remained in CR.

Eight of the surviving patients had limited cGvHD (one of them had disease relapse) and one patient suffered from extensive cGvHD. Of note, this patient still had a very favorable quality of life despite immunosuppressive therapy.

These observations underline growing evidence that allogeneic HCT can be safely performed even in elderly patients and can lead to stable CR in many patients^11^ resulting in OS at 24 months of 39% and relapse rates of 55%.^12^

Previous studies in elderly patients that also included patients <70 years showed favorable 2-year survival rates of up to 50%^13^ and low GvHD incidences as low as 9%.^2^ Here, incidence of aGvHD and cGVHD was 29% and 32%, respectively. Therefore, incidence of aGvHD was higher compared with our previous observations in patients ⩾60 years. Although the incidence of severe GvHD has been reported to increase with older age,^14^ the incidence of cGvHD tended to be lower as compared with other observations in patients ⩾60 years reporting 52%.^13^ Of note, patients ⩾70 years were reported to have similar incidences of 34% overall cGvHD.^12^ It has to be noted that in the largest cohort of elderly patients, cumulative incidence of NRM (31%) was much higher and OS was distinctly lower (25%).^1^ This might be due to the fact that these patients were treated between 1998 and 2008. Hence, recent development of more elaborate RIC regimens might lead to lower incidences of NRM and favorable OS.

In contrast to other studies, we here for the first time provide data that may identify elderly patients that could benefit more from an HCT: patients with high (but not very high)-risk diseases, CR before HCT, short intervals between diagnosis and HCT, HCT from UD and maybe male sex. Moreover, our data indicate that immunosuppressive agents might contribute to the prognosis. However, owing to the limited patient number potentially confounding factors could not be excluded in multivariate analyses. Therefore, the prospective evaluation of the most suitable immunosuppression is required.

Although these observations derive from a retrospective analysis, our results indicate that HCT can be a curative treatment option even for patients ⩾70 years. Owing to the use of RIC, there is a higher risk of relapse as compared with younger patients also with a significant GvHD morbidity. Although we are not able to compare with a matched control group who were treated with cytoreductive therapy or best supportive care, our data are more favorable as historical results in AML patients treated with Decitabine indicated by a comparable median OS of 7.7 months but less favorable long-term survival of 18% in the study of Kantarjian *et al.*^15^ In summary, our data warrant a prospective evaluation of allogeneic HCT in a homogenous elderly population to define specific subpopulations, especially with regard to donor selection, immunosuppression and risk stratification. Allogeneic HCT in qualifying patients ⩾70 years should be considered as a curative treatment option to accomplish sufficient patient numbers for evaluation of these subgroups. Today, precise selection and sufficient education of the respective elderly patients are essential.

## Figures and Tables

**Figure 1:**
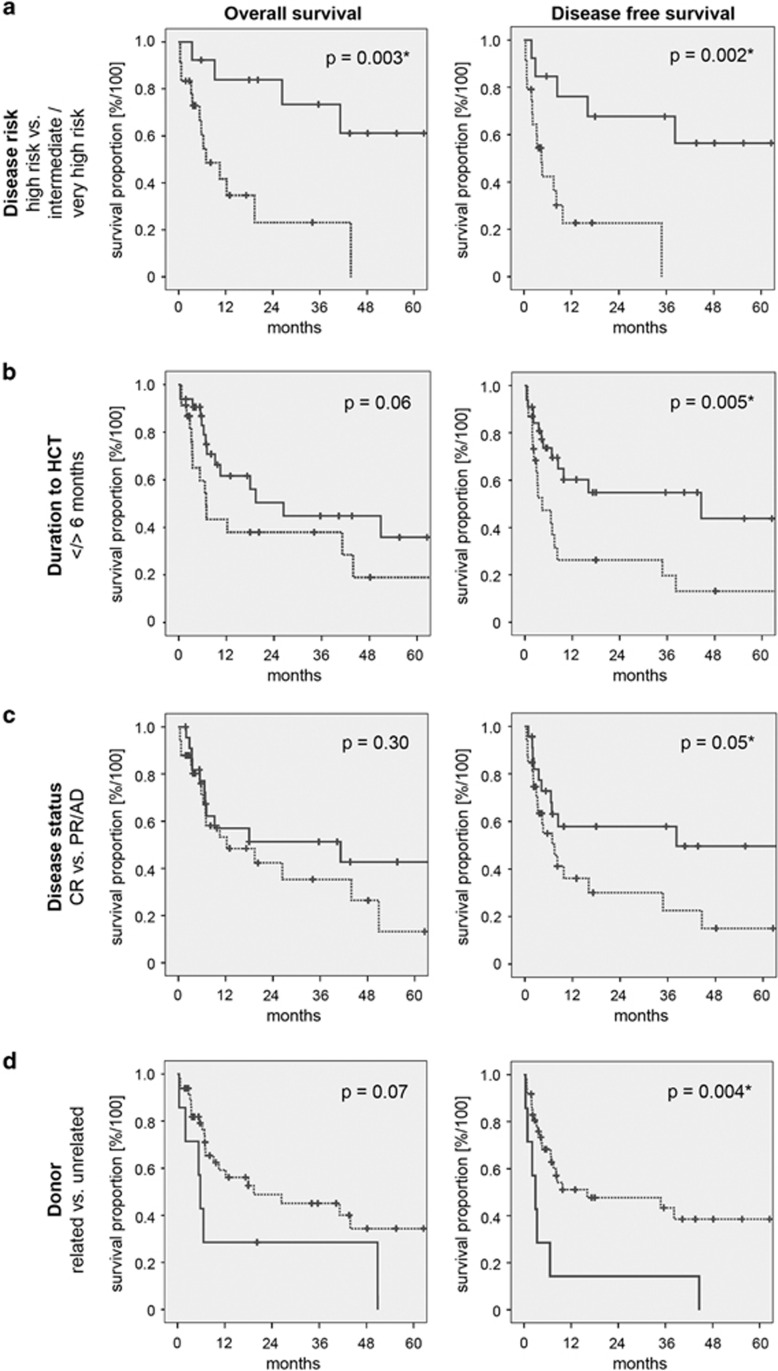
Subgroup analyses using Kaplan–Meier survival estimates for OS and DFS. OS (left panels) and DFS (right panels) analyses are shown for relevant patient subgroups with statistical significant differences. (**a)** OS and DFS stratified according to disease risk (high/intermediate risk, solid line; very high risk, dotted line). (**b)** OS and DFS stratified according to the time interval between initial diagnosis and HCT (< 6 months, solid line; >6 months, dotted line). (**c)** OS and DFS stratified according to disease status at HCT (CR, solid line; PR or AD, dotted line). (**d)** OS and DFS stratified according to donor relatedness (related donor, solid line; UD, dotted line).

**Table 1:** Patient characteristics and clinical results

*Patients*		
Patients	*n*=56	
Women	*n*=22	39%
Men	*n*=34	61%
Median age (years)	71	Range 70–79
		
*Diagnoses*		
AML	*n*=46	82%
CLL	*n*=1	2%
MDS	*n*=5	9%
NHL	*n*=1	2%
PMF	*n*=3	5%
		
*Comorbidities*		
a) HCT-CI^a^		
Median	1	Range 0–10
0	*n*=14	25%
1–2	*n*=22	39%
⩾ 3	*n*=20	36%
b) Integrated NRM score^a^		
Median	5	Range 2–12
0–3	*n*=7	12%
4–6	*n*=28	50%
⩾ 7	*n*=21	38%
		
*Risk group (DRI)*		
Low	*n*=19	34%
Intermediate	*n*=10	18%
High	*n*=13	23%
Very high	*n*=14	25%
*Transplantation*		
*Time to HCT*^b^		
Median (months)	5	Range 1–190
		
*Disease stage at HCT*		
CR	*n*=23	41%
PR	*n*=15	27%
Active disease	*n*=18	32%
		
*Donors*		
MRD	*n*=7	13%
MUD	*n*=37	66%
MMUD	*n*=12	21%
CMV mismatch	*n*=16	29%
Blood-type mismatch	*n*=32	57%
		
*Conditioning regimen*		
RIC	*n*=56	100%
		
*Stem cell source*		
PBSC	*n*=55	98%
BM	*n*=1	2%
		
*CD34*^*+*^ *cells in graft*		
Median	6.54x10^6^/kg BW	Range 1.95–18.04x10^6^/kg BW
*Outcome*		
*Engraftment*		
No engraftment	*n*=4	7%
Neutrophils (>500/μl)	Median day 19	Range days 9–43
Platelets (>25 000/μl)	Median day 15	Range days 10–398
		
*GvHD*		
Acute	*n*=16	29%
Median	Grade 1	Range 1–4
Chronic	*n*=18	32%
Limited	*n*=13	23%
Extensive	*n*=5	9%
		
*OS*^c^		
Median (months)	18.0	Range 0.4–123.9
1-Year OS	54.7%	
2-Year OS	46.1%	
3-Year OS	42.8%	
5-Year OS	18.7%	
Causes of death	*n*=29	100%
Relapse	*n*=20	69%
Infection	*n*=3	10%
GvHD	*n*=2	7%
Hemorrhage	*n*=2	7%
Embolism	*n*=1	3%
Graft failure	*n*=1	3%
NRM	*n*=9	16%
*DFS*^d^		
Median (months)	8.4	Range 0.4–123.9
Relapse	*n*=22	39%
Median time to relapse (months)	3.9	Range 0.8–44.6

Abbreviations: AML, acute myeloid leukemia; BM, bone marrow; BW, bodyweight; CLL, chronic lymphoid leukemia; CMV, cytomegalovirus; CR, complete remission; DFS, disease-free survival; DRI, disease risk index; GvHD, graft versus host disease; HCT, hematopoietic cell transplantation; HCT-CI, HCT-related comorbidity index; MDS, myelodysplastic syndrome; MMUD, mismatched unrelated donor; MRD, matched related donor; MUD, matched unrelated donor; NHL, non-Hodgkin lymphoma; NRM, non-relapse mortality; OS, overall survival; PBSC, peripheral blood stem cell; PMF, primary myelofibrosis; PR, partial remission; RIC, reduced intensity conditioning.

a For a detailed overview of factors contributing to HCT-CI and integrated NRM score, please refer to Supplementary Table 1.

b Time between initial diagnosis and HCT.

c Time between HCT and death of any cause or last follow-up visit. Patients alive at last follow-up visit were censored.

d Time from HCT until relapse. Patients without relapse were censored at the last day of follow-up or on death.
