# Supplementary material for: Allogeneic hematopoietic cell transplantation in patients ⩾70 years: which patients may benefit?
Source: Blood Cancer J. 2016 Jul 8;6(7):e443–. doi: 10.1038/bcj.2016.54 (PMC5030379; doi:10.1038/bcj.2016.54)
Supplement: Supplementary Figure 1 [file bcj201654x1.pdf]

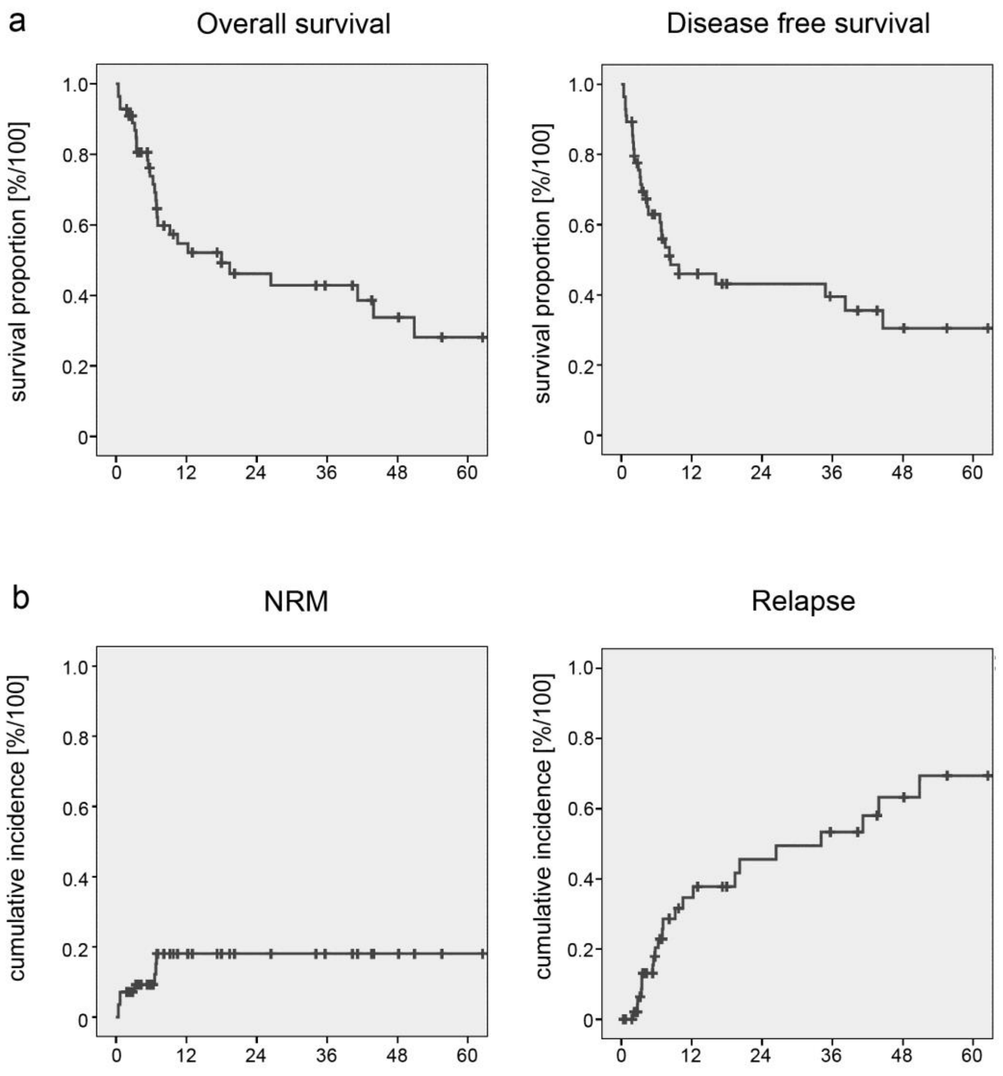

Supplementary Figure 1: Kaplan-Meier survival estimates, cumulative incidence and COX-regression analyses.

a OS (left panel) and DFS (right panel) for the whole patient cohort. **b** Cumulative incidence of NRM adjusted for competitive risk progression (left panel) and cumulative incidence of disease relapse adjusted to competitive risk NRM (right panel).
