# Supplementary material for: Allogeneic hematopoietic cell transplantation in patients ⩾70 years: which patients may benefit?
Source: Blood Cancer J. 2016 Jul 8;6(7):e443–. doi: 10.1038/bcj.2016.54 (PMC5030379; doi:10.1038/bcj.2016.54)
Supplement: Supplementary Table 1 [file bcj201654x2.pdf]

**Comorbidities**		
Factors integrated in HCT-CI and/or NRM score		
Age > 60 years	n = 56	100 %
Arrhythmia	n = 6	11 %
Cardiac disease	n = 10	18 %
Cerebrovascular disease	n = 4	7 %
CIBD	n = 0	0 %
Diabetes	n = 5	9 %
Hepatic disease	n = 0	0 %
Infection	n = 2	4 %
Obesity	n = 1	2 %
Peptic ulcer	n = 3	5 %
Previous malignant disease	n = 16	29 %
Psychiatric disturbance	n = 3	5 %
Pulmonary disease	n = 6	11%
Renal Disease	n = 0	0 %
Rheumatologic disease	n = 8	14 %
Valvular heart disease	n = 4	7 %
MUD	n = 37	66 %
Donor CMV	n = 25	45 %
Recipient CMV	n = 36	64 %
Time interval to HCT > 6 months	n = 23	41 %
**Conditioning Regimen**		
Fludarabin / Busulfan	n = 26	46 %
Fludarabin / 2 Gy TBI	n = 12	21 %
Fludarabin / Melphalan	n = 1	2 %
Fludarabin / BCNU / Melphalan	n = 2	4 %
Fludarabin / Treosulfan / Rituximab	n = 1	2 %
FLAMSA / Fludarabin / Busulfan	n = 12	21 %
FLAMSA / Busulfan / Cyclophosphamide	n = 1	2 %
Ibritumomab-Tiuxetan / Fludarabin / Melphalan	n = 1	2 %
**Immunosuppression**		
Ciclosporin A based	n = 12	21 %
+ Alemtuzumab	n = 2	4 %
+ ATG + MMF	n = 4	7 %
+ MMF	n = 4	7 %
+ MMF + Sirolimus	n = 2	4 %
Tacrolimus based	n = 42	75 %
+ ATG + MTX	n = 21	38 %
+ ATG + MMF	n = 14	25 %
+ MMF	n = 4	7 %
+ MMF + Sirolimus	n = 2	4 %
+ MTX	n = 1	2 %

Methotrexate based		
+ ATG	n = 1	2 %
Sirolimus based		
+ ATG	n = 1	2 %

Supplementary table 1: Additional patient and transplantation characteristics
Abbreviations: ATG: anti-thymocyte globulin; BM: bone marrow; MRD: matched related donor; MMF: mycophenolate mofetil; MMUD: mismatched unrelated donor; MTX: methotrexate; MUD: matched unrelated donor; NMA: non myeloablative conditioning; PBSC: peripheral blood stem cell; RIC: reduced intensity conditioning.
