# Supplementary material for: Allogeneic hematopoietic cell transplantation in patients ⩾70 years: which patients may benefit?
Source: Blood Cancer J. 2016 Jul 8;6(7):e443–. doi: 10.1038/bcj.2016.54 (PMC5030379; doi:10.1038/bcj.2016.54)
Supplement: Supplementary Table 2 [file bcj201654x3.pdf]

OS	Median	KM-estimate 3-year OS % (95% CoI)		LogRank	Hazard Ratio
**Sex**					
female	10.5	26 %	(3.5 – 17.5)	p = 0.06	0.50 (0.24 – 1.05)
male	50.9	58 %			
**Disease risk**					
intermediate/very high	7.0	23 %	(1.3 – 12.7)	p = 0.003*	5.11 (1.59 – 16.49)
high	70.4	73 %			
low/intermediate	7.1	35 %	(0.0 – 21.9)	p = 0.27	0.66 (0.31 – 1.38)
high/very high	41.2	51 %	(0.0 – 86.0)		
**Duration to HCT**					
< 6 months	26.4	45 %	(10.4 – 42.4)	p = 0.06	1.98 (0.95 – 4.12)
> 6 months	6.9	38 %	(4.6 – 9.2)		
**Disease status at HCT**					
CR	41.2	51 %	(0.0 – 94.8)	p = 0.3	1.49 (0.70 – 3.20)
PR/AD	12.3	35 %	(0.0 – 28.3)		
**Donor**					
related	5.8	29 %	(4.7 – 6.9)	p = 0.07	0.44 (0.18 – 1.09)
unrelated	19.4	45 %	(0.0 – 49.8)		
**Immunosuppression**					
no CSA	10.5	31 %	(4.0 – 17.0)	p = 0.09	0.44 (0.16 – 1.19)
CSA	43.9	74 %	(10.8 – 77.0)		
ATG	19.4	41 %	(1.9 – 36.9)	p = 0.57	0.80 (0.37 – 1.73)
no ATG	6.8	44 %	(1.7 – 11.9)		
Alemtuzumab	0.4	0 %		p = 0.003*	6.93 (1.53 – 31.38)
no Alemtuzumab	19.4	45 %	(0.0 – 39.5)		
**HCT-CI**					
< 3	12.3	38 %	(0.0 – 26.0)	p = 0.83	0.92 (0.43 – 1.98)
> 3	43.9	51 %	(0.0 – 101.0)		
**Blood group**					
match	19.4	41 %	(5.3 – 33.5)	p = 0.73	1.14 (0.55 – 2.38)
mismatch	10.5	44 %	(3.2 – 17.8)		
**Donor**					
matched	10.5	46 %	(0.0 – 46.2)	p = 0.88	1.07 (0.45 – 2.54)
mismatched	18.0	36 %	(0.0 – 36.9)		
**CMV**					
match	18.0	38 %	(3.2 – 32.8)	p = 0.74	0.87 (0.38 – 1.96)
mismatch	50.9	55 %	(0.4 – 101.3)		

Supplementary Table 2: Subgroup Analyses for overall survival. Statistical significant values are marked with an asterisk (*).
Abbreviations: AD: active disease; ATG: anti-thymocyte globulin; CI: comorbidity index; CoI: confidence interval; CMV: cytomegalovirus; CoI: Confidence Interval; CR: complete remission; CSA: ciclosporin A; DFS: disease free survival; HCT: hematopoietic cell transplantation; KM: Kaplan-Meyer; OS: overall survival; PR: partial remission.
