# Supplementary material for: Allogeneic hematopoietic cell transplantation in patients ⩾70 years: which patients may benefit?
Source: Blood Cancer J. 2016 Jul 8;6(7):e443–. doi: 10.1038/bcj.2016.54 (PMC5030379; doi:10.1038/bcj.2016.54)
Supplement: Supplementary Table 3 [file bcj201654x4.pdf]

DFS	Median	KM-estimate 3-year DFS % (95% CoI)		LogRank	Hazard Ratio
**Sex**					
female	8.4	26 %	(4.3 – 12.5)	p = 0.38	0.73 (0.36 – 1.48)
male	44.6	52 %	(0.0 – 107.3)		
**Disease risk**					
intermediate/very high	4.4	0 %	(2.6 – 6.2)	p = 0.002*	5.22 (1.65 – 16.54)
high	70.4	68 %			
low/intermediate	6.8	32 %	(3.4 – 10.2)	p = 0.42	0.75 (0.37 – 1.52)
high/very high	16.1	48 %	(0.0 – 50.3)		
**Duration to HCT**					
< 6 months	44.6	55 %	(0.0 – 97.5)	p = 0.005*	2.69 (1.31 – 5.51)
> 6 months	4.4	20 %	(0.0 – 9.1)		
**Disease status at HCT**					
CR	38.2	58 %	(0.0 – 97.0)	p = 0.05*	2.11 (0.98 – 4.53)
PR/AD	7.5	23 %	(2.4 – 12.6)		
**Donor**					
related	2.9	14 %	(0.8 – 5.0)	p = 0.004*	0.31 (0.13 – 0.72)
unrelated	16.1	43 %	(0.0 – 47.3)		
**Immunosuppression**					
no CSA	6.9	32 %	(4.9 – 8.9)	p = 0.09	0.44 (0.17 – 1.18)
CSA	38.2	63 %	(0.0 – 78.1)		
ATG	9.8	42 %	(0.0 – 21.4)	p = 0.76	0.89 (0.42 – 1.88)
no ATG	6.8	37 %	(3.3 – 10.3)		
Alemtuzumab	0.4	0 %		p < 0.001*	10.44 (2.20 – 49.55)
no Alemtuzumab	9.8	41 %	(0.0 – 21.6)		
**HCT-CI**					
< 3	8.4	39 %	(5.1 – 11.7)	p = 0.91	1.04 (0.50 – 2.16)
> 3	6.8	40 %	(0.0 – 42.3)		
**Blood group**					
match	16.1	47 %	(0.0 – 56.8	p = 0.68	1.17 (0.57 – 2.40)
mismatch	8.2	35 %	(2.6 – 13.8)		
**Donor**					
matched	8.4	45 %	(0.0 – 42.4)	p = 0.91	0.95 (0.41 – 2.24)
mismatched	16.1	20 %	(3.1 – 29.1)		
**CMV**					
match	8.4	37 %	(0.0 – 20.6)	p = 0.80	1.10 (0.52 – 2.35)
mismatch	9.8	43 %	(4.0 – 15.6)		

Supplementary Table 3: Subgroup Analyses for disease free survival. Statistical significant values are marked with an asterisk (*).

Abbreviations: AD: active disease; ATG: anti-thymocyte globulin; CI: comorbidity index; CoI: confidence interval; CMV: cytomegalovirus; CoI: Confidence Interval; CR: complete remission; CSA: ciclosporin A; DFS: disease free survival; HCT: hematopoietic cell transplantation; KM: Kaplan-Meyer; OS: overall survival; PR: partial remission.
