# Supplementary material for: Allogeneic hematopoietic cell transplantation in patients ⩾70 years: which patients may benefit?
Source: Blood Cancer J. 2016 Jul 8;6(7):e443–. doi: 10.1038/bcj.2016.54 (PMC5030379; doi:10.1038/bcj.2016.54)
Supplement: Supplementary Table 4 [file bcj201654x5.pdf]

Patients	Number	Percentage of surviving patients (n = 27)	Percentage of initial cohort (respective characteristics)
**Surviving Patients**	n = 27	100 %	48 % (27/56)
Women	n = 5	19 %	23 % (5/22)
Men	n = 22	81 %	65 % (22/34)
Median age	72 years	range 70-77 years	
HCT-CI	median 1	range 0-10	
Integrated NRM score	median 6	range 2-12	
**Risk group**			
Low	n = 9	33 %	47 % (9/19)
Intermediate	n = 3	11 %	30 % (3/10)
High	n = 8	30 %	62 % (8/13)
Very high	n = 7	26 %	50 % (7/14)
**Time to HCT**			
< 6 months	n = 19	70 %	58 % (19/33)
> 6 months	n = 8	30 %	35 % (8/23)
**Disease Stage at HCT**			
CR	n = 11	41 %	48 % (11/23)
PR	n = 9	33 %	60 % (9/15)
AD	n = 7	26 %	38 % (7/18)
**Donors**			
MRD	n = 1	4 %	14 % (1/7)
MUD	n = 21	77 %	57 % (21/37)
MMUD	n = 5	19 %	42 % (5/12)
**CMV mismatch**	n = 8	30 %	50 % (8/16)
**Blood group mismatch**	n = 16	59 %	50 % (16/32)
**Stem cell source**			
PBSC	n = 26	96 %	47 % (26/55)
BM	n = 1	4 %	100 % (1/1)
**$CD34^+$ cells in graft**	median 6.45×10^6 / kg BW	range $1.95 – 13.8 \times 10^6$ / kg BW	median (deceased patients): 7.4×10^6 / kg BW $p = 0.5$

Supplementary table 4: Patient and transplantation characteristics of the surviving patients (n = 12).

Abbreviations: AD: active disease; BM: bone marrow; CMV: cytomegalovirus; CR: complete remission; MRD: matched related donor; MMUD: mismatched unrelated donor; MUD: matched unrelated donor; NMA: non myeloablative conditioning; PBSC: peripheral blood stem cell; PR: partial remission; RIC: reduced intensity conditioning.
